# Increasing the resolution of the reconstructed image in terahertz pulse time-domain holography

**DOI:** 10.1038/s41598-018-36642-3

**Published:** 2019-01-17

**Authors:** Nikolay S. Balbekin, Maksim S. Kulya, Andrey V. Belashov, Andrei Gorodetsky, Nikolay V. Petrov

**Affiliations:** 1 ITMO University, Saint-Petersburg, 197101 Russia; 2 Ioffe Institute, St. Petersburg, 194021 Russia; 3 Department of Chemistry, Imperial College London, London, SW7 2AZ UK

## Abstract

In this paper, we present a novel numerical approach for increasing the resolution of retrieved images of objects after their diffraction patterns are recorded via terahertz pulse time-domain holography (THz PTDH). THz PTDH allows for spectrally resolved imaging with high spatial resolution and does not require the fine alignment of complex optics in the THz path. The proposed data post-processing method opens up the possibility to reconstruct holograms recorded with spatially restricted THz detectors, and overcome the diffraction limit even for the lower-frequency spectral components. The method involves an iterative procedure of backward-forward wavefront propagation to simulate the field distribution beyond the initially recorded hologram area. We show significant improvement in both the object reconstruction and contrast across the whole spectrum, with qualitative resolution enhancement at lower frequency spectral components.

## Introduction

Since its first demonstration back in 1980s^1^, terahertz (THz) radiation has found numerous spectroscopic and imaging applications^2^. With the development of compact yet powerful broadband THz transceivers^3^, THz time-domain spectroscopy (THz TDS) has become a vital tool for numerous diagnostic tasks. Although it can be time consuming, point-by-point object scanning with the focused THz TDS beam allows for spectrally resolved THz imaging. This approach, first demonstrated in 1996^4,5^, has been successfully applied to numerous applications, including: (i) *in vitro* and *in vivo* tissue characterization on the cellular level for ophtalmology^6^, dentistry^7,8^ and cancerous cell detection^9–13^; (ii) non-contact monitoring of parcels and luggage contents for concealed metals or chemical/biological threats^14–17^; (iii) quality control and identification of harmful substances in food products^18–20^; (iv) defect testing in materials^21–25^; (v) chemical composition analysis for oils and fuels^26–28^; and (vi) non-destructive composition analysis of high-value art objects and antiquities^29–32^. All of these examples involve point-by-point scanning with THz TDS setups, and would greatly benefit from immediate detection of transverse complex amplitude spatio-temporal distribution in the wavefront cross-section.

THz imaging with matrix detectors usually involves the use of narrowband sources, gas lasers^33–35^, free electron lasers^36^, or (most popularly nowadays) quantum cascade lasers (QCLs)^37^, with incoherent detection of intensity distribution by microbolometer^34,37^ or pyroelectric^33,35,36^ arrays. Although this type of imaging allows for amplitude and phase image reconstruction, it does not provide any crucial spectroscopic data. By contrast, THz Pulse Time-Domain Holography (THz PTDH)^38,39^ yields full wavefront reconstruction for all spectral components, thus providing immediate information on matter distribution across a larger area cross-section after a single time-domain scan and consecutive numerical reconstruction.

Earlier, a way to detect the whole THz wavefront coherently was demonstrated^40^, and later applied for pioneering pulsed THz non-holographic^41^ and holographic reconstruction^38,42–44^. However, there are still many unsolved problems in THz PTDH, some of them being fundamental. THz PTDH allows one to obtain a high resolution in all three dimensions. Nevertheless, the capabilities of the method are limited by relatively small measurement areas, because of the detection sensitivity and low availability of the large electro-optic crystals. To overcome the aforementioned limitations, several approaches have been proposed in monochromatic CW THz holography: several exposures of the hologram, while moving the detector^34^, numerical filtering for signal-to-noise ration enhancement^45^, and iterative phase retrieval method^33,46,47^.

From traditional holography in the visible range, it is well known that an object can be retrieved from an arbitrary small hologram, with resolution inversely proportional to its size^48^. In lensless digital holography, an additional restriction is set by the number of pixels in the digitized hologram. The hologram size and number of pixels are even more vital in THz holography. On one hand, the sensor area cannot be immediately extended to cover a wider spatial angle, while on the other hand, the pixel size cannot be reduced since it is already equal or less than THz wavelengths, reaching from ∼100 µm to ∼3 mm.

Recently, for visible range holography, Latyshevskaia *et al*. proposed a self-extrapolation technique^49^ which allows for overcoming the Abbe criterion and resulting resolution enhancement of the reconstructed objects by padding the hologram and iteratively filling the padded area with the numerically propagated wavefront. Theoretical justification (more detailed in^50^) of the complex wave spatial extrapolation was discussed by J.L. Harris^51^, who has shown the uniqueness of the problem solution for given spatial spectrum distribution of the analytical signal on the basis of Whittaker Watson law “the spectrum of a size-limited object is always analytical”^52^ and Guellemin law “a function of a complex variable is determined throughout the entire Z-plane from a knowledge of its properties within an arbitrarily small region of analyticity”^53^ Later, this method was extended to phase retrieval^50,54^ and inline narrowband THz holography^33^.

In this paper, we propose an adaptation of the above mentioned technique for resolution enhancement of broadband THz pulse time-domain holograms. This method is in high demand, due to the absence of large (in the numbers of wavelength) area THz detectors. We show that the enhancement of the holograms occurs only at wavelengths that yield at least one full interference fringe within the recorded hologram. To the best of our knowledge, this is the first demonstration of the method for broadband radiation.

## Terahertz Pulse Time-Domain Holography

To record THz pulse time domain hologram one must first record the amplitude and phase of a collimated wavefront at the detection plane, then place the object of interest to record the diffraction pattern with the same detector. As a matter of fact it is a broadband inline holography with coherent detection provided by pulsed THz that gives information on both amplitude and phase distribution upon measurement.

**Figure 1:**
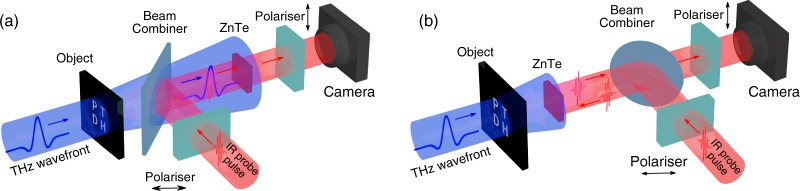
Setups for THz pulse time domain holography with codirectional (**a**) and retroreflected (**b**) probe IR beams.

There are two main techniques for coherent THz detection: (i) free-space electro-optical sampling (EOS)^55^ or; (ii) detection in photoconductive antennas (PCAs)^1^. Each technique has its own advantages and disadvantages^56^, but they can both be implemented in synchronous and asynchronous^57,58^ layouts, providing reasonably comparable results, the latter being more fast but at the same time requiring significantly more expensive setup. The PTDH resolution enhancement approach we offer can be applied to any coherent wavefront THz detector, and, as there are currently no array PCA detectors, we propose the following layouts for full wavefront EOS using either a synchronous, or asynchronous approach (Fig. 1).

The setup on the left is based on a classical EOS THz detection layout. The more advanced setup on the right uses a retroreflected probe IR beam, thus allowing for closer object-to-hologram plane distance, and therefore, a larger spatial angle that positively affects the resolution. Although the full wavefront is detected in both layouts, the size of the hologram is constrained by size of both the matrix detector and the electro-optic crystal. The detection sensitivity also imposes a limit on the hologram size, since commercially-available coherent broadband THz transmitters are not powerful enough to be effectively detected in larger cross-sections.

Consider the object ${\mathscr{O}}(x,y,{\rm{\nu }})$ placed in the THz wavefront. After diffraction on the object, the incoming THz field $E(x,y,t)$ is distorted into ${E}_{{\mathscr{O}}}(x,y,t)$. Further propagation to the registration plane at the distance *l* forms the diffraction pattern ${E}_{ {\mathcal H} }(\tilde{x},\tilde{y},t^{\prime} )$. By applying a Fourier transform, this pulse wavefront can be converted into the frequency domain

1 $$ {\mathcal H} (\tilde{x},\tilde{y},{\rm{\nu }})={\int }_{-\infty }^{+\infty }{E}_{ {\mathcal H} }(\tilde{x},\tilde{y},t^{\prime} )\exp (\,-\,i2\pi \nu t^{\prime} )dt^{\prime} $$

${\mathcal H} (\tilde{x},\tilde{y},{\rm{\nu }})$ is the Fourier transform of the hologram recorded in the time domain at the distance *L* from the object plane, and can be considered as a union of monochromatic holograms calculated for every frequency *v*.

**Figure 2:**
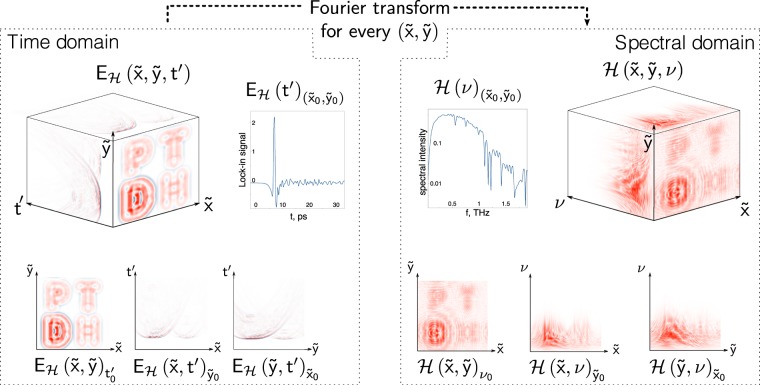
Three-dimensional data array transformation from temporal to complex frequency representation. The representation of data as 2D arrays.

Generally, we can consider the set of independent monochromatic objects ${\mathscr{O}}(x,y,{\rm{\nu }})$ spatially digitized into *M* × *M* pixels to lead to a transverse size of *D* = (*M*Δ*x*, *M*Δ*y*), corresponding to pixel size of Δ*x* × Δ*y*. This set of objects produces a set of independent holograms ${\mathcal H} (\tilde{x},\tilde{y},{\rm{\nu }})$, each being *N* × *N* pixels and the overall transverse size of $\tilde{D}=(N{\rm{\Delta }}\tilde{x},\,N{\rm{\Delta }}\tilde{y})$. The number of both sub-objects and sub-holograms in the sets is equal to the number of frequency components in the spectrum^42^. Likewise, both object and hologram can be represented as sets of spatial-temporal field distributions, or spatial-spectral intensity charts. This concept is depicted in Fig. 2.

The THz hologram is a three-dimensional array of data recorded after diffraction on an object. This array carries information about the spatial-temporal, or spatial-spectral distribution of the THz field.

To reconstruct an object from a hologram, the recorded complex field is back-propagated into the object plane:

2 $${\mathscr{O}}^{\prime} (x,y,{\rm{\nu }})={ {\mathcal F} }_{L,{\rm{\nu }}}^{-1}[ {\mathcal H} (\tilde{x},\tilde{y},{\rm{\nu }})],$$

where ${ {\mathcal F} }_{L,\nu }^{-1}[{\mathbb{H}}]\to {\mathbb{O}}$ is the inverse wavefront propagation operator, here, ${\mathbb{H}}$ and ${\mathbb{O}}$ are the sets of all holograms and objects, correspondingly. Depending on the distance between the object and the hologram, it can be either a Rayleigh-Sommerfeld integral, 2D Fourier or 2D Fresnel transforms, or angular spectrum calculation^39,59^.

## Iterative self-extrapolation algorithm for PTDH

**Figure 3:**
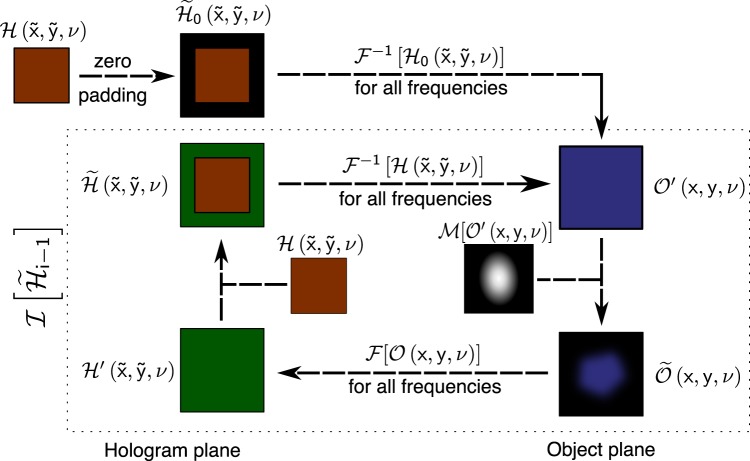
The scheme of the algorithm of extrapolation of the spatial distribution of the field beyond the hologram registration area.

The principle for object self-healing in the case of broadband THz PTDH is quite similar to the one described for monochromatic holography^49^. Proposed technique is based on the assumption that the diffraction pattern, recorded in the hologram, is created by waves that exist not only within, but also outside the registration area. Therefore, self-extrapolation of the digital hologram to the area outside of the experimentally recorded diffraction pattern can be performed. The principal scheme for the algorithm in the case of broadband THz PTDH is shown in Fig. 3. In general words, the hologram is back-propagated to reconstruct an object from the data ‘as is’, as described in equation (2). Then, after the application of the mask $\tilde{{\mathscr{O}}}(x,y,{\rm{\nu }})={ {\mathcal M} }_{x,y}[{\mathscr{O}}^{\prime} (x,y,{\rm{\nu }})]$ that restricts the energy distribution in the object plane and helps to avoid the ghost image appearance, the reconstructed object wavefront is numerically propagated to form a synthesized hologram

3 $$ {\mathcal H} ^{\prime} (\tilde{x},\tilde{y},{\rm{\nu }})={ {\mathcal F} }_{L,\nu }[\tilde{{\mathscr{O}}}(x,y,{\rm{\nu }})],$$

${\mathcal F} [{\mathbb{O}}]\to {\mathbb{H}}$ is the forward propagation operator, that, similarly to inverse operator ${ {\mathcal F} }^{-1}$ is chosen depending on the distance between object and hologram. Thus, we can introduce an iteration operator ${\mathcal I} [{\mathbb{H}}]\to {\mathbb{H}}$, describing the reconstruction of an object $\tilde{{\mathscr{O}}}$, and consecutive generation of a synthesized hologram $\tilde{ {\mathcal H} }^{\prime}$ as follows:

4 $$ {\mathcal I} ({\mathbb{H}})\to {\mathbb{H}}: {\mathcal I} ( {\mathcal H} )= {\mathcal F} \,[ {\mathcal M} [{ {\mathcal F} }^{-1}[ {\mathcal H} ]]]\mathrm{.}$$

When this operator is applied, one should keep in mind that any region of the object plane that is involved in calculations, but does not contain meaningful information, must be zeroed to avoid ghost images^60^. While calculation, it is achieved by multiplication of the recovered object by the mask ${ {\mathcal M} }_{(x,y)}[{\mathscr{O}}]$ that removes all unwanted areas by zeroing them.

The iterative algorithm can be described by the following scheme: if it is a zero-step, zeropad the recorded hologram ${\mathcal H} (\tilde{x},\tilde{y},{\rm{\nu }})$ to achieve a larger area wavefront in the hologram plane ${\tilde{ {\mathcal H} }}_{0}(\tilde{x},\tilde{y},{\rm{\nu }})$, for other steps, replace the central part of the synthesized hologram ${ {\mathcal H} }_{i}^{^{\prime} }(\tilde{x},\tilde{y},{\rm{\nu }})= {\mathcal I} [{\tilde{ {\mathcal H} }}_{i-1}(\tilde{x},\tilde{y},{\rm{\nu }})]$, corresponding to originally recorded hologram $({\rm{rect}}(\frac{\tilde{x}}{N{\rm{\Delta }}\tilde{x}},\frac{\tilde{y}}{N{\rm{\Delta }}\tilde{y}}) > 0)$, with the originally recorded hologram ${\mathcal H} (\tilde{x},\tilde{y},{\rm{\nu }})$:

5 $${\tilde{ {\mathcal H} }}_{i}(\tilde{x},\tilde{y},{\rm{\nu }})=\{\begin{array}{l} {\mathcal H} (\tilde{x},\tilde{y},{\rm{\nu }}),\,{\rm{rect}}(\frac{\tilde{x}}{N{\rm{\Delta }}\tilde{x}},\frac{\tilde{y}}{N{\rm{\Delta }}\tilde{y}}) > 0\\  {\mathcal I} [{\tilde{ {\mathcal H} }}_{i-1}(\tilde{x},\tilde{y},{\rm{\nu }})],\,{\rm{rect}}(\frac{\tilde{x}}{N{\rm{\Delta }}\tilde{x}},\frac{\tilde{y}}{N{\rm{\Delta }}\tilde{y}})=0\,\cap \,i > 0\\ 0,\,{\rm{rect}}(\frac{\tilde{x}}{N{\rm{\Delta }}\tilde{x}},\frac{\tilde{y}}{N{\rm{\Delta }}\tilde{y}})=0\,\cap \,i=0\end{array}$$

After a reasonable number of iterations, the reconstructed object is recovered, along with its spectral features.

## Results and Discussion

Since the spectrum of THz pulsed radiation is more that 1 octave wide, depending on the object type, the algorithm can be implemented in a different manner. For spectrally uniform objects, like binary amplitude opaque (e.g. metal) objects, reconstructed data obtained with one of the wavelengths can be used as a synthetic object in the next iteration for forward propagation simulation with a different wavelength. Thus, an enhancement across the whole spectrum can be achieved. On the other hand, for an object with numerous spectral features, each feature can be reconstructed independently, provided that the numerical aperture of the hologram gives the sufficient amount of wavefront data for reconstruction. Indeed, if the full contrast interference fringe is not recorded, the object will not be retrieved^49^.

### Image reconstruction by low frequency components of the THz range

Let us consider the problem of increasing the resolution of a binary amplitude object. In accordance with the Abbe criterion, the resolution of the reconstructed image for each spectral component is limited by the numerical aperture *NA* of the hologram, hence it depends on the distance between the object and the registration plane, as well as the linear dimensions of the hologram as

6 $${R}_{Abbe}=\frac{c}{2\cdot {\rm{\nu }}\cdot NA}\approx \frac{cl}{{\rm{\nu }}D},$$

where *R*_*Abbe*_ is the resolution in m, c is the speed of light, *D* and *l* are the transverse size and distance to the hologram. Thus, after a size restricted hologram ${ {\mathcal H} }_{0}(\tilde{x},\tilde{y},{\rm{\nu }})$ is recorded, application of the iterative algorithm and hologram self extrapolation obtains the field ${\tilde{ {\mathcal H} }}_{i}(\tilde{x},\tilde{y},{\rm{\nu }})$, which has a larger transverse size, thus enhancing the reconstructed image resolution.

**Figure 4:**
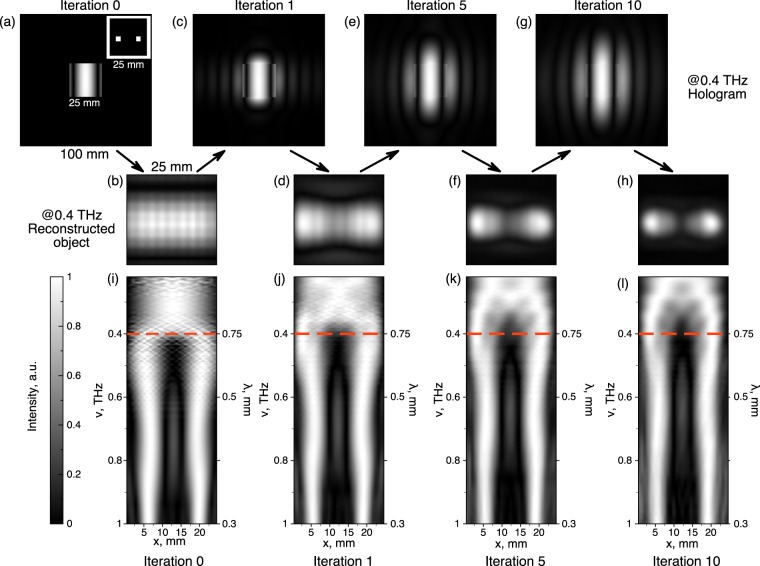
The illustration of the algorithm application to the broadband THz PTDH.

Figure 4 illustrates the results of the algorithm applied to the binary object of the two apertures separated by ∼15 mm. The object itself, 25 × 25 mm^2^ in size, is shown in the inset of Fig. 4(a). The central part of Fig. 4(a) shows the simulated 25 × 25 mm^2^ hologram at 0.4 THz, recorded at 600 mm from the object. This frequency is selected, as in this frequency range the algorithm application demonstrates the highest effect. Indeed, if we calculate the initial resolution at 0.4 THz, it is equal to ${R}_{0}^{{\rm{A}}bbe}=18$ mm, and the result of the object reconstruction from this hologram is shown in Fig. 4(b). Then, this reconstruction is used to simulate the hologram ${\tilde{ {\mathcal H} }}_{1}(\tilde{x},\tilde{y},{\rm{\nu }})$ shown in Fig. 4(c), with its central part replaced with initially recorded ${ {\mathcal H} }_{0}(\tilde{x},\tilde{y},{\rm{\nu }})$, as it was described in Fig. 3. The wavefront in this hologram is wider than the area of the initially recorded hologram, hence the *NA* and the reconstruction resolution is higher (${R}_{1}^{{\rm{A}}bbe}=4.5$ mm). The reconstruction after first iterative procedure shown in Fig. 4(d), already reveals the start of the two dots separation. After the 10th algorithm iteration, the theoretical resolution reaches ${R}_{10}^{{\rm{A}}bbe}=3$ mm, and the two dots in the reconstruction are clearly resolved. Holograms and reconstructed objects at further iteration steps (5th and 10th), are shown in Fig. 4(e,g) and (f,h), respectively.

Spectra of the reconstructed images cross-sections (Fig. 4(i–l)) demonstrate the effectiveness of the proposed method. While slightly enhancing the reconstructed images in the higher frequency range, the crucial change is revealed in the spectral area between 0.35–0.45 THz (670–850 *μm*), where the wavelength is approximately equal to the detector pixel size (780 *μm*) and the theoretical resolution changes from ${R}_{0}^{{\rm{A}}bbe}\approx 23$ mm to ${R}_{10}^{{\rm{A}}bbe}\approx 3$ mm upon iterations, i.e. from below to above the dots separation, and the dots become resolvable. At higher frequencies, the dots are being resolved even after the direct hologram reconstruction, and at longer wavelengths, the *NA* of the hologram is still not enough to cover at least one interference fringe and thus provide enough contrast in the object reconstruction. As a result, the apertures, although they are larger than the wavelength, can not be recovered through the proposed algorithm. Such a fast convergence of the algorithm, as compared with results in^49^, occurs due to longer THz wavelengths involved (hence smaller objects, holograms, and distances between them in wavelength units) and higher impact of every iteration step. Note that reconstruction of the image at the frequency of 0.42 THz require only 10 iteration, while at lower frequencies (e.g. 0.38 THz) more iterations should be performed for significant improvement of reconstructed image spatial resolution.

### Application of the technique to spectrally selective object reconstruction

**Figure 5:**
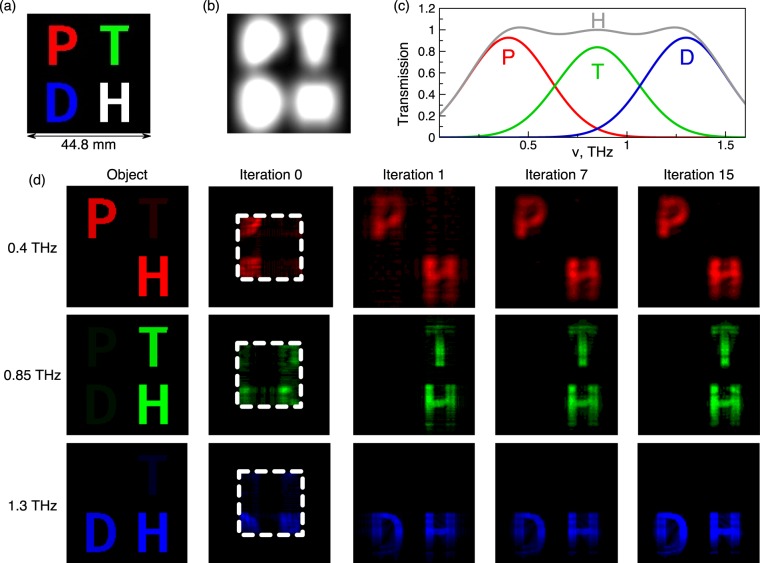
Input data for modeling the reconstruction of a spectrally selective spatially inhomogeneous object. The object of investigation (**a**), mask (**b**), performing a mathematical constraint, the transmission spectrum of regions of a non-uniform object (**c**). Reconstructed images of the “PTDH” object at different wavelengths without after 0th, 1st, 7th and 15th iterations (**d**). White frame indicates the size of the recorded hologram.

Next, we study a spectrally selective spatially inhomogeneous object (Fig. 5). As an object, we used opaque mask 44.8 × 44.8 mm^2^ in size with ‘P’, ‘T, ‘D’ and ‘H’ letters transparent in different spectral windows.

The spectral-selective amplitude transmission of an object is given by the function:

7 $$\begin{array}{rcl}T(x,y) & = & R(x,y)\exp [-{(\frac{{\rm{\Delta }}{\rm{\nu }}-r}{{{\rm{\Delta }}{\rm{\nu }}}_{r}})}^{2}]\\  &  & +\,G(x,y)\exp [-{(\frac{{\rm{\Delta }}{\rm{\nu }}-g}{{{\rm{\Delta }}{\rm{\nu }}}_{g}})}^{2}]\\  &  & +\,B(x,y)\exp [-{(\frac{{\rm{\Delta }}{\rm{\nu }}-b}{{{\rm{\Delta }}{\rm{\nu }}}_{b}})}^{2}]\end{array}$$

Here *R*(*x*, *y*), *G*(*x*, *y*) and *B*(*x*, *y*) are spatial normalization coefficients of the amplitude for the Gaussian distribution; ${{\rm{\nu }}}_{r},{{\rm{\nu }}}_{g},{{\rm{\nu }}}_{b}$ are fixed central frequencies of the normal distribution 0.4 THz, 0.85 THz and 1.3 THz respectively; ${{\rm{\Delta }}{\rm{\nu }}}_{r}$, ${{\rm{\Delta }}{\rm{\nu }}}_{g}$, ${{\rm{\Delta }}{\rm{\nu }}}_{b}$ are average standard deviations from fixed frequencies ${{\rm{\nu }}}_{r},{{\rm{\nu }}}_{g},{{\rm{\nu }}}_{b}$ respectively. The transmission spectra are shown in Fig. 5(c). The letters P, T and D in Fig. 5(a) are transparent in the lower (red), medium (green) and high (blue) frequency ranges, respectively. Meanwhile, the letter H is transparent in all three frequency ranges. The size of the square object in Fig. 5(a) is 44.8 × 44.8 mm^2^. The size of the simulated broadband time-domain hologram (marked on the figure by the white dashed square) is 22.4 × 22.4 mm^2^, and the diffraction pattern was simulated 100 mm away from the object using a flat broadband wavefront. Figure 5(d) and Table 1 present the results of the iterative object reconstruction. Both spectral and spatial features eventually get resolved after ∼10 iterations, and the object gains larger area of fine reconstruction, which initially was restricted by the smaller size of the hologram and very low scattering introduced by the objects. As a result, the mean root square error (MRSE) of reconstruction quality (normalized error between reconstructed image compared to initial object) drops from 0.66 to 0.34 (see Table 1). Due to the fact that MRSE depends mostly on the general shape of the object rather than on fine structures of the object (in this case sharp edges of the letters), significant decrease of MRSE can be observed right after the first iteration, which provides overall shape of the letters. On the other hand, addition of higher spatial frequency components during further iterations, which results in increase of letter edge reconstruction quality, does not introduce significant contribution to decrease of MRSE.

**Table 1:** Results of MRSE calculation.

Frequency, THz	0th iteration	1st iteration	7th iteration	15th iteration
0.4	0.66	0.39	0.36	0.37
0.85	0.53	0.36	0.34	0.34
1.3	0.58	0.39	0.38	0.37

## Conclusion

In this paper, we proposed the solution for resolution and field of view enhancement in THz pulse time domain holography (THz PTDH). THz PTDH allows for spectrally resolved images to be obtained without any additional optics in the THz beam, as the signal is coherently detected after diffraction on the object of study. The resolution enhancement method involves an iterative procedure of backward-forward wavefront propagation, with numerical extension of the hologram area. The method is especially important for THz holography, as the detectors are usually restricted in size, and the relatively large wavelength means that a very low number of interference fringes can be recorded. We have shown the method to enhance both the resolution and the field of view for the reconstructed images after performing only 10 iterations. We successfully applied the method for both spectrally uniform and spectrally selective objects, and revealed significant enhancement at all frequencies that produce at least one interference fringe in the diffraction pattern of the recorded hologram. At the frequencies between 0.35–0.45 THz, the Abbe criterion for the object and hologram under study is reduced from the value of 23 mm (above the distance between the apertures used as an object) at the first iteration, to the value of 3 mm (well below the distance between the apertures) at the last iteration. Spectrally selective test object also revealed significant reconstruction quality enhancement resulting in double reduction of reconstruction mean root square error. Thus, the proposed method can be can be successfully applied to all kinds of pulsed THz holographic imaging tasks, a well as to spatio-temporal metrology of broadband complex wavefronts^59,61^. Field of view broadening, in all these tasks, when THz detection is performed with relatively small crystal, will push the boundaries of the method experimental applicability.

## Methods

Numerical simulations were performed with in-house software, written in National Instruments LabView development framework. The software follows mathematical procedures described in this paper, and our preceeding works^39,59,61^.

## References

[1] Auston DH, Cheung KP, Smith PR (1984). Picosecond photoconducting Hertzian dipoles. Appl. Phys. Lett..

[2] Jepsen P, Cooke D, Koch M (2011). Terahertz spectroscopy and imaging - Modern techniques and applications. Laser Photon. Rev.

[3] Lepeshov S, Gorodetsky A, Krasnok A, Rafailov E, Belov P (2017). Enhancement of terahertz photoconductive antenna operation by optical nanoantennas. Laser Photon. Rev.

[4] Hu BB, Nuss MC (1995). Imaging with terahertz waves. Opt. Lett..

[5] Chan WL, Deibel J, Mittleman DM (2007). Imaging with terahertz radiation. Reports on Progress in Physics.

[6] Bennett DB (2011). Terahertz sensing in corneal tissues. Journal of Biomedical Optics.

[7] Crawley D (2003). Three-dimensional terahertz pulse imaging of dental tissue. Journal of Biomedical Optics.

[8] Smirnov SV, Grachev YV, Tsypkin AN, Bespalov VG (2014). Experimental studies of the possibilities of diagnosing caries in the solid tissues of a tooth by means of terahertz radiation. J. Opt. Technol.

[9] Yu C, Fan S, Sun Y, Pickwell-Macpherson E (2012). The potential of terahertz imaging for cancer diagnosis: A review of investigations to date. QIMS.

[10] Ruth MW (2002). Terahertz pulse imaging in reflection geometry of human skin cancer and skin tissue. Phys. Med. Biol..

[11] Fitzgerald A (2006). Terahertz Pulsed Imaging of human breast tumors. Radiology.

[12] Wallace VP (2004). Terahertz pulsed imaging of basal cell carcinoma *ex vivo* and *in vivo*. British Journal of Dermatology.

[13] Smolyanskaya, O. *et al*. Terahertz biophotonics as a tool for studies of dielectric and spectral properties of biological tissues and liquids. *Prog. Quantum Electron*., 10.1016/j.pquantelec.2018.10.001 (2018).

[14] Federici JF (2005). THz imaging and sensing for security applications—explosives, weapons and drugs. Semiconductor Science and Technology.

[15] Shen YC (2005). Detection and identification of explosives using terahertz pulsed spectroscopic imaging. Applied Physics Letters.

[16] Kemp MC (2011). Explosives Detection by Terahertz Spectroscopy—A Bridge Too Far?. IEEE T. THz Sci. Techn.

[17] Zhang, L., Zhong, H., Deng, C., Zhang, C. & Zhao, Y. Terahertz wave reference-free phase imaging for identification of explosives. *Applied Physics Letters* **92** (2008).

[18] Gowen A, O’Sullivan C, O’Donnell C (2012). Terahertz time domain spectroscopy and imaging: Emerging techniques for food process monitoring and quality control. Trends in Food Science & Technology.

[19] Ok G, Park K, Kim HJ, Chun HS, Choi S-W (2014). High-speed terahertz imaging toward food quality inspection. Applied Optics.

[20] Massaouti M, Daskalaki C, Gorodetsky A, Koulouklidis AD, Tzortzakis S (2013). Detection of Harmful Residues in Honey Using Terahertz Time-Domain Spectroscopy. Appl. Spectrosc..

[21] Stoik CD, Bohn MJ, Blackshire JL (2008). Nondestructive evaluation of aircraft composites using transmissive terahertz time domain spectroscopy. Optics Express.

[22] Balbekin, N. S., Novoselov, E. V., Pavlov, P. V., Bespalov, V. G. & Petrov, N. V. Nondestructive monitoring of aircraft composites using terahertz radiation. In *Proc. of SPIE*, vol. 9448, 94482D (2015).

[23] Ospald F (2013). Aeronautics composite material inspection with a terahertz time-domain spectroscopy system. Optical Engineering.

[24] Amenabar I, Lopez F, Mendikute A (2013). In introductory review to THz non-destructive testing of composite mater. Journal of Infrared, Millimeter, and Terahertz Waves.

[25] Yakovlev EV, Zaytsev KI, Dolganova IN, Yurchenko SO (2015). Non-Destructive Evaluation of Polymer Composite Materials at the Manufacturing Stage Using Terahertz Pulsed Spectroscopy. IEEE T. THz Sci. Techn..

[26] Zhao H, Zhao K, Bao R (2012). Fuel property determination of biodiesel-diesel blends by terahertz spectrum. J. Infrared Millim. Te.

[27] Yin M, Tang S, Tong M (2016). The application of terahertz spectroscopy to liquid petrochemicals detection: A review. Applied Spectroscopy Reviews.

[28] Watanabe Y (2003). Component spatial pattern analysis of chemicals using terahertz spectroscopic imaging. Applied Physics Letters.

[29] Skryl AS, Jackson JB, Bakunov MI, Menu M, Mourou GA (2014). Terahertz time-domain imaging of hidden defects in wooden artworks: application to a Russian icon painting. Applied Optics.

[30] Jackson JB (2014). Terahertz pulse imaging in archaeology. Frontiers of Optoelectronics.

[31] Öhrström L (2015). Terahertz Imaging Modalities of Ancient Egyptian Mummified Objects and of a Naturally Mummified Rat. Anatomical Record.

[32] Krügener K (2015). Terahertz meets sculptural and architectural art: Evaluation and conservation of stone objects with t-ray technology. Sci. Rep.

[33] Rong L (2014). Terahertz in-line digital holography of dragonfly hindwing: amplitude and phase reconstruction at enhanced resolution by extrapolation. Opt. Express.

[34] Zolliker P, Hack E (2015). THz holography in reflection using a high resolution microbolometer array. Optics express.

[35] Rong L (2015). Terahertz in-line digital holography of human hepatocellular carcinoma tissue. Scientific reports.

[36] Choporova YY, Knyazev BA, Mitkov MS (2015). Classical Holography in the Terahertz Range: Recording and Reconstruction Techniques. IEEE Trans. Terahertz Sci. Technol..

[37] Hack E, Zolliker P (2014). Terahertz holography for imaging amplitude and phase objects. Opt. Express.

[38] Gorodetsky AA, Bespalov VG (2010). THz pulse time-domain holography. Proc. of SPIE.

[39] Petrov NV, Kulya MS, Tsypkin AN, Bespalov VG, Gorodetsky A (2016). Application of terahertz pulse time-domain holography for phase imaging. IEEE T. THz Sci. Techn.

[40] Wu Q, Hewitt T, Zhang X-C (1996). Two-dimensional electro-optic imaging of THz beams. Applied Physics Letters.

[41] Ushakov A, Chizhov P, Bukin V, Savel’ev A, Garnov S (2018). Broadband in-line terahertz 2D imaging: comparative study with time-of-flight, cross-correlation, and Fourier transform data processing. J. Opt. Soc. Am. B.

[42] Bespalov VG, Gorodetsky A (2007). Modeling of referenceless holographic recording and reconstruction of images by means of pulsed terahertz radiation. Journal of Optical Technology.

[43] Zhang Y, Zhou W, Wang X, Cui Y, Sun W (2008). Terahertz Digital Holography. Strain.

[44] Wang X, Cui Y, Sun W, Zhang Y (2012). Terahertz digital holography. Proc. of SPIE.

[45] Chen G, Li Q (2015). Markov chain Monte Carlo sampling based terahertz holography image denoising. Applied Optics.

[46] Petrov, N. V., Gorodetsky, A. & Bespalov, V. G. Holography and phase retrieval in terahertz imaging. In *Proc. SPIE*, **8846**, 88460S (2013).

[47] Petrov, N. V., Bespalov, V. G. & Volkov, M. V. Phase retrieval of THz radiation using set of 2D spatial intensity measurements with different wavelengths. In *Proc. of SPIE*, **8281**, 82810J–82810J–7 (2012).

[48] Gabor D (1949). Microscopy by Reconstructed Wave-Fronts. Proceedings of the Royal Society A: Mathematical, Physical and Engineering Sciences.

[49] Latychevskaia T, Fink H-W (2013). Resolution enhancement in digital holography by self-extrapolation of holograms. Optics Express.

[50] Latychevskaia T, Chushkin Y, Fink HW (2016). Resolution enhancement by extrapolation of coherent diffraction images: a quantitative study on the limits and a numerical study of nonbinary and phase objects. Journal of Microscopy.

[51] Harris JL (1964). Diffraction and resolving power. JOSA.

[52] Whittaker, E. T. & Watson, G. N. *A course of modern analysis* (Cambridge university press, 1996).

[53] Guillemin, E. A. *The mathematics of circuit analysis* (John Wiley New York, 1949).

[54] Katkovnik V, Shevkunov I, Petrov NV, Egiazarian K (2017). Computational super-resolution phase retrieval from multiple phase-coded diffraction patterns: simulation study and experiments. Optica.

[55] Wu Q, Zhang X (1995). Free-space electro-optic sampling of terahertz beams. Applied Physics Letters.

[56] Grachev YV, Osipova MO, Bespalov VG (2014). Comparison of an electro-optical system and photo-conducting antenna employed as detectors of pulsed terahertz radiation by means of a new method for measuring spectral width. Quantum Electronics.

[57] Yasui T, Saneyoshi E, Araki T (2005). Asynchronous optical sampling terahertz time-domain spectroscopy for ultrahigh spectral resolution and rapid data acquisition. Applied Physics Letters.

[58] Brown MS (2006). Water-vapor detection using asynchronous THz sampling. Appl. Spectrosc..

[59] Kulya MS, Semenova VA, Bespalov VG, Petrov NV (2018). On terahertz pulsed broadband gauss-bessel beam free-space propagation. Sci. Rep.

[60] Latychevskaia, T. & Fink, H. W. Solution to the twin image problem in holography. *Physical Review Letters* **98** (2007). 10.1103/PhysRevLett.98.233901 17677906

[61] Kulya, M., Semenova, V., Gorodetsky, A., Bespalov, V. G. & Petrov, N. V. Spatio-temporal and spatio-spectral metrology of terahertz broadband uniformly topologically charged vortex beams. *Applied Optics* **58**(5), (2019). 10.1364/AO.58.000A90 30873965

